# Obesity and hypertension in children and adolescents

**DOI:** 10.1186/s40885-024-00278-5

**Published:** 2024-09-01

**Authors:** Soo In Jeong, Sung Hye Kim

**Affiliations:** 1 Department of Pediatrics, Ajou University Hospital, Ajou University School of Medicine, Suwon, Republic of Korea; 2 Department of Pediatrics, CHA Bundang Medical Center, CHA University, 59 Yatap-ro, Bundang-gu, Seongnam-si, Gyeonggido, Republic of Korea

**Keywords:** Hypertension, Pediatric obesity, Children and adolescents, Treatment

## Abstract

As childhood obesity rates increase worldwide, the prevalence of obesity-related hypertension is also on the rise. Obesity has been identified as a significant risk factor for hypertension in this age group. National Health Surveys and meta-analyses show increasing trends in obesity and pediatric hypertension in obese children. The diagnosis of hypertension in children involves percentiles relative to age, sex, and height, unlike in adults, where absolute values are considered. Elevated blood pressure (BP) in childhood is consistently associated with cardiovascular disease in adulthood, emphasizing the need for early detection and intervention. The pathogenesis of hypertension in obesity involves multiple factors, including increased sympathetic nervous system activity, activation of the renin-angiotensin-aldosterone system (RAAS), and renal compression due to fat accumulation. Obesity disrupts normal RAAS suppression and contributes to impaired pressure natriuresis and sodium retention, which are critical factors in the development of hypertension. Risk factors for hypertension in obesity include degree, duration, and distribution of obesity, patient age, hormonal changes during puberty, high-sodium diet, sedentary lifestyle, and socioeconomic status. Treatment involves lifestyle changes, with weight loss being crucial to lowering BP. Medications such as angiotensin-converting enzyme inhibitors or angiotensin II receptor blockers may be considered first, and surgical approaches may be an option for severe obesity, requiring tailored antihypertensive medications that consider individual pathophysiology to avoid exacerbating insulin resistance and dyslipidemia.

## Background

Hypertension is one of the most important chronic diseases associated with severe cardiovascular complications. The diagnosis and treatment of hypertension in children and adolescents is essential because it is associated with hypertension and metabolic syndrome in adults. While hypertension in children was previously characterized by secondary hypertension, primary hypertension is becoming an important cause in many countries with increasing numbers of overweight and obese children. In particular, obesity is a significant risk factor for hypertension in this age group.

The authors aim to explore the prevalence of hypertension, its association with obesity, the mechanisms by which it develops in obese children, and the treatment of hypertension in obese children and adolescents.

### Hypertension definition, hypertension trajectory, hypertension prevalence, and trends

Unlike in adults, the diagnosis of hypertension in children and adolescents is defined as being above the 95th percentile of the distribution by sex, age, and height, not the absolute value [1]. In addition, the European Society of Hypertension proposes to diagnose hypertension using the same criteria as adults aged 16 years and older [2], and the American Academy of Pediatrics (AAP) suggests diagnosing hypertension using the same criteria as adults in those 13 years and older [3]. In recent years, there has been a movement toward more uncomplicated diagnoses of hypertension, with the Canadian Society of Hypertension adding to the previous definition to define hypertension as > 120/80 mmHg in 6- to 11-year-olds and 130/85 mmHg in 12- to 17-year-olds [4]. Japan also reintroduced absolute values for specific age groups (Table 1) [5]. Hypertension is diagnosed when blood pressure (BP) is elevated on three or more visits and can be measured using mercury sphygmomanometers, nonmercury aneroid sphygmomanometers, and oscillating sphygmomanometers, with confirmation recommended by aneroid auscultatory sphygmomanometers [1–4].

**Table 1:** Various definitions of hypertension in children and adolescents

Guideline	SBP and/or DBP Percentile of Category					
**TFR** [1]	Normal	Prehypertension	Hypertension			
	< 90th	90th to < 95th of if BP exceeds 120/80 even if < 90th percentile up to < 95th percentile	≥ 95th percentile			
**ESH** [2]	1–15 yr			≥ 16 years		
	Normal	High-normal	Hypertension	Normal	High-normal	Hypertension
	< 90th percentile	≥ 90th percentile to < 95th percentile	≥ 95th percentile	< 130/85 mmHg	130–139/85–89 mmHg	≥ 140/90 mmHg
**AAP** [3]	1–13 yr			≥ 13 yr		
	Normal	Elevated BP	Hypertension	Normal	Elevated BP	Hypertension
	< 90th percentile	≥ 90th percentile to < 95th percentile or 12/80 mmHg to < 95th percentile (whichever is lower)	≥ 95th percentile	< 120/80 mmHg	120/<80 to 129/<80 mmHg	≥ 130/80 mmHg
	**Criteria for hypertension**					
**Canadian** [4]	6–11 years:			12–17 years		
	≥ 95th or ≥ 120/80 mmHg (whichever is lower)			≥ 95th or ≥ 130/85 mmHg (whichever is lower)		
**Japan** [5]	Pre-School	Elementary 1 ~ 3rd grade	Elementary 4 ~ 6 grade	Junior high boys	Junior high girls	High school
	≥ 120/70 mmHg	≥ 130/80 mmHg	≥ 135/80 mmHg	≥ 140/85 mmHg	≥ 135/80 mmHg	≥ 140/85 mmHg

*AAP* American Academy of Pediatrics, *BP* blood pressure, *DBP* diastolic blood pressure, *ESH*, European Society of Heart, *SBP* systolic blood pressure, *TFR* The Fourth Report

Studies have consistently shown that high BP in children contributes to cardiovascular disease in adults [6–9]. In the Atherosclerosis Risk in Healthy Young Adults study, systolic BP (SBP) at a mean age of 13 years was associated with intima-media thickening in adulthood [6]. In the Young Finns Study, which followed 2204 people for 27 years, BP, serum lipid levels, and body mass index (BMI) in childhood were also strongly associated with those in middle age [7]. The Coronary Artery Risk Development in Young Adults Study, which followed 4681 people for 25 years, found that the risk of a coronary artery calcium score of 100 HU or more at 25 years was significantly higher in the group with increased BP than those with low, controlled BP [8]. A cohort study that followed 975 people aged 7 to 38 also found that those with elevated BP had worse cardiovascular outcomes, demonstrating the importance of early detection, prevention, and intervention [9].

According to the Korea National Health and Nutrition Examination Survey (KNHANES), SBP showed an increasing trend between 2007 and 2015, and this trend was particularly pronounced in obese children. The prevalence of hypertension also increased from 6.9% in 2007–2009 to 9.0% in 2013–2015, with a significant increase from 14.9 to 27.7% in obese children [10]. According to the China Health and Nutrition Survey, BP and the prevalence of hypertension showed an increasing trend between 1991 and 2015, with hypertension increasing from 8.5% in 1991 to 19.2% in 2015 [11]. Factors specifically associated with hypertension included adolescents aged 13–17 years (OR = 1.76), general obesity (OR = 2.69), and central obesity (OR = 1.49). The prevalence of hypertension in children and adolescents aged 12–19 years based on data from the National Health and Nutrition Examination Survey (NHANES) in the United States, on the other hand, decreased somewhat in 2013–2016 compared to 2001–2004, from 7.7 to 4.2% under the 2017 AAP guidelines [12]. However, the prevalence of obesity increased from 17.8 to 21.8% during this period, and the prevalence of hypertension among obese children was 9.43% during 2013–2016, which is higher than the prevalence of hypertension among all children [12]. Meanwhile, a study based on a school-based BP screening program reported 16.3% elevated BP and 2.7% hypertension among students aged 10–17 years. In particular, 10.6% had stage 1 hypertension at initial screening, but only 2.7% had confirmed hypertension after two additional visits, demonstrating the importance of repeat visits and BP measurements in pediatric hypertension [13]. In the meta-analysis of 47 articles, the global pooled prevalence of hypertension was 4.0% (95% confidence interval (CI), 3.29-4.78%), and that of prehypertension was 9.67% (95% CI, 7.26-12.38%) (Table 2) [14]. In addition, pediatric hypertension has shown an increasing trend over the past two decades in this analysis. However, another systematic review analyzed 18 studies of changes in childhood hypertension between 1963 and 2012 and found that BP gradually decreased in 13 studies, increased in four, and remained unchanged in one [15].

**Table 2:** The prevalence of hypertension in the various studies

Study	Age group (year)	Year	Hypertension prevalence	Hypertension prevalence in obesity	Data source
Cho et al. [10]	10–18	2007–2009	6.9%	14.9%	KNHANES
		2013–2015	9.0%	27.7%	
Ye et al. [11]	7–17	1991	8.5%		The China Health and Nutrition Survey
		2015	19.2%		
Jackson et al. [12]*	12–19	2001	7.7%		NHANES
		2016	4.2%	9.43%	
Bell et al. [13] ^†^	10–17	2000–2017	2.7%		The Houston Pediatric and Hypertension Program
Song et al. [14]	-19		4.0%	15.27%	Meta-analysis
		1990s	1.26%		
		2000s	3.30%		
		2010–2014	6.02%		

*KNHANES* Korea National Health and Nutrition Examination Survey, *NHANES* National Health and Nutrition Examination Survey

* The prevalence of hypertension defined by the 2017 AAP guideline

^†^ The prevalence of hypertension defined by the Fourth Report

### Obesity definition, obesity prevalence and trends

Obesity in children and adolescents is defined by BMI, which is calculated by dividing weight by the square of height. This calculated BMI is then compared to normal for age and gender, with overweight defined as between the 85th percentile and 95th percentile, obesity defined as above the 95th percentile, and severe obesity defined as above 120% of the 95th percentile or above 35 kg/m^2^ [16]. The AAP further defines severe obesity as BMI ≥ 120% to < 140% of the 95th percentile or BMI ≥ 35 to < 40 kg/m^2^ as class 2 obesity and BMI ≥ 140% of the 95th percentile or BMI ≥ 40 kg/m^2^ as class 3 obesity [17].

The World Health Organization reports that globally, 33 million children under the age of 5 years [5.3% (UI: 5.1–5.5)] were classified as overweight in 2000, rising to 37 million [5.6% (UI: 5.1–6.1)] by 2022 [18]. In 2416 population-based studies, from 1975 to 2016, the global age-standardized prevalence of obesity among children and adolescents aged 5 to 19 years in 200 countries increased from 0.7% (95% credible index 0.4–1.2) in 1975 to 5.6% (4.8–6.5) in 2016 for girls and from 0.9% (0.5–1.3) to 7.8% (6.7–9.1) for boys [19]. Trends in BMI change varied by region, flattening somewhat in Northwest Europe and high-income countries but increasing for both sexes in East and South Asia. The number of obese girls grew from 5 million in 1975 to 50 million in 2016, while the number of obese boys grew from 6 million to 74 million during the same period. 73% of this increase is attributable to the rise in obese children [19]. According to the 2017–2018 NHANES in the United States, 19.3% of children aged 2–19 years were obese, 6.1% of whom were severely obese, and 16.1% were overweight. This is a significant increase compared to 1971–1974, when 5.2% were obese [20]. The prevalence of obesity among children and adolescents in Korea is also increasing based on data from the National School Health Examination (NSHE) and the KNHANES, from 8.7% in 2007 to 15.0% in 2017 according to the NSHE data and from 8.6% in 2001 to 9.8% in 2017 according to the KNHANES data. This increase was particularly pronounced among boys and high school students (Table 3) [21].

**Table 3:** The prevalence of obesity in the various studies

Study	Age group (year)	Region	Year	Obesity prevalence	Data source
NCD Risk Factor Collaboration [19]	5–19	Worldwide	1975	0.7% (girls) 0.9% (boys)	A pooled analysis of 2416 population-based measurement studies
			2016	5.6% (girls) 7.8% (boys)	
Fryar et al. [20]	2–19	USA	1971–1974	5.2%	NHANES
			2017–2018	19.3%	
Kim et al. [21]	6–18	Korea	2007	8.7%	NSHE
			2017	15.0%	
	2–18	Korea	2001	8.6%	KNHANES
			2017	9.8%	

*KNHANES* Korea National Health and Nutrition Examination Survey, *NCD* non-communicable diseases, *NHANES* National Health and Nutrition Examination Survey, *NSHE* National School Health Examination

The relationship between BP and BMI has been shown in several studies [22, 23]. In a study comparing 167 obese adolescents with 31 nonobese youth, SBP was significantly higher, and elevated BP was substantially more common in those with a BMI Z score of 2.5 or greater compared to those with a BMI Z score of less than 2 [22]. In another study of BP and BMI associations in adolescents, mean SBP and diastolic BP (DBP) were correlated with increasing BMI deciles, in which an increase in BMI from the 1st to the 10th decile was associated with a 10 mmHg increase in SBP and a 3–4 mmHg increase in DBP [23]. In children, excess weight can be a risk factor for later BP. When 17,816 students were followed for 8.2 years, the risk of hypertension was 1.53 times higher for boys and 1.28 times higher for girls if their BMI remained persistently high compared with those whose BMI remained persistently low [24]. Additionally, several studies have shown a higher prevalence of hypertension in overweight or obese children. In a school-based screening of 5102 children, the prevalence of hypertension was 2% for BMI below the 5th percentile, increasing to 11% for those above the 95th percentile, with overweight being a significant relative risk for hypertension [25]. Data from the NHANES of the United States showed that the prevalence of hypertension according to the 2017 AAP guidelines was 4.2% among all children aged 12–19 years from 2013 to 2016, compared to 9.43% among obese children and 14.7% among severely obese children [12]. In a study of 57,915 overweight and obese children aged 6–18 years from 188 centers in Germany, Austria, and Switzerland, the prevalence of hypertension and prehypertension ranged from 27 to 47%, depending on the hypertension criteria [26].

### Pathogenesis of hypertension in obesity

Overweight and obesity affect BP in many ways, and adiposity and weight gain are essential contributors to primary hypertension [27]. The development of hypertension in obesity is influenced by several factors, including increased sympathetic nervous system (SNS) activity, activation of the renin-angiotensin-aldosterone system (RAAS), and compression of the kidneys due to fat accumulation, resulting in increased renal sodium reabsorption and impaired pressure natriuresis.

Increased SNS is thought to play an essential role as a mechanism for hypertension in obesity. In a study showing that weight gain itself increases SNS, 12 healthy men were made to gain 5 kg of weight by overeating, and when comparing before and after, there was a significant increase in total fat and abdominal fat and a substantial increase in muscle SNS and SBP, suggesting a relationship between obesity and hypertension [28]. In a study of 18 lean and 25 overweight healthy college students, overweight was associated with subclinical alterations in renal and endothelial function in addition to left ventricular wall thickness regardless of hypertension [29]. SNS activity was highly associated with cardiovascular and renal changes in these individuals. In obesity, not all SNSs are increased, but the kidneys and muscles seem to play an important role [30]. The fat distribution also plays an important role, with increased muscle SNS activity observed in individuals with visceral adiposity alone [31]. SNS overactivity is also associated with ethnicity. Native American Pima Indians, who have very high rates of obesity, have a relatively low frequency of hypertension. They have increased adiposity and insulinemia but have lower basal muscle SNS activity than white individuals and a lower frequency of hypertension [32]. In addition, sleep apnea, which is common in obesity, causes chronic hypoxia, which activates chemoreceptors in the carotid body and upregulates SNS activity [33].

Mediators of increased SNS activity include hyperinsulinemia, angiotensin II, impairment of baroreceptor reflexes, activation of chemoreceptor-mediated reflexes associated with sleep apnea, and cytokines such as leptin, tumor necrosis factor-a, and interleukin-6 secreted by adipocytes. Of these, leptin is secreted by adipocytes and is particularly elevated in obesity and hypertension. In one study, normotension was observed in children with severe obesity and a leptin gene mutation, and in these children, SNS activity was actually decreased [34]. In addition, leptin is selectively resistant in obesity, resulting in a reduced appetite-reducing effect but a preserved SNS activity response in the kidneys [35].

Despite the volume expansion and sodium retention associated with obesity, they do not have a normal RAAS suppressive response. Related factors, such as plasma renin activity, angiotensinogen, angiotensin-converting enzyme (ACE), and aldosterone, are elevated compared to normal subjects [36, 37]. Renin secretion is also upregulated in obese patients under the pressure of increased visceral and retroperitoneal fat. Adipocytes from subcutaneous fat are also an essential source of angiotensin II and can downregulate the RAAS in subcutaneous adipocytes by regulating insulin [38]. In obese patients, RAAS and SNS activity interact to stimulate renin secretion.

In obesity, visceral, retroperitoneal, and renal sinus fat cause renal compression and structural changes in kidney tissue. Physical compression by fat in and around the kidneys impairs pressure natriuresis and increases renal tubular sodium reabsorption, resulting in sodium retention and increased BP [39–41]. Obesity increases the glomerular filtration rate and effective renal plasma flow but ultimately leads to glomerular injury, which increases BP and causes renal damage, creating a deleterious cycle [39, 40].

### Risk factors for hypertension in obesity

Obesity is the most critical risk factor for hypertension in children and adolescents [42, 43]. Risk factors other than obesity that are known to contribute to the development of hypertension include male sex, a family history of hypertension, early life factors such as birth weight or gestational age, a high-sodium diet, the absence of a Dietary Approaches to Stop Hypertension (DASH)-type diet, larger amounts of sedentary time, and possibly other dietary factors [3, 44–46]. However, it is not yet clear what risk factors for hypertension are specific to overweight children and adolescents.

Factors related to the characteristics of obesity concerning the development of hypertension include the degree, duration, and distribution of obesity and the patient’s age. In a study conducted by Babinska et al. on 109 obese children aged 7–18 years with a BMI Z score above 1.65, only 24% had normal BP on ambulatory BP monitoring, while 3% had hypertension, and 48% had severe ambulatory hypertension [47]. The study also found a proportional association between BMI and the severity of ambulatory hypertension and daytime BP. In adults, abdominal obesity is a significant predictive factor for hypertension. Chen et al. conducted a cross-sectional study on the correlation between the types of obesity and hypertension in adult males in the United States using NNHANES data from 2007 to 2018 and found that the presence of abdominal obesity was significant in predicting hypertension compared to BMI alone [48]. The association of the waist-to-height ratio (WHtR), one of the measurements of abdominal obesity in children and adolescents, with hypertension varies across studies but is generally significant [49–52]. In a meta-analysis of nine studies involving 25,424 children aged 6 to 18, waist circumference (WC) and WHtR did not show superiority in detecting elevated BP [50]. In another meta-analysis including 21 cross-sectional studies involving 177,943 children aged 3–19, BMI, WC, and WHtR showed no significant difference in predicting hypertension and elevated BP [51]. However, in a cross-sectional study involving Malaysian adolescents between 12 and 16 years, WHtR presented good sensitivity and specificity in males and females [52]. Kułaga et al. investigated abdominal obesity cutoffs associated with adult cardiovascular risk thresholds based on anthropometric data from Polish children aged 3–18 years and found that the determined abdominal obesity cutoff significantly predicted hypertension and elevated BP [53]. The duration of obesity is also crucial in the development of hypertension in obese children. In a cross-sectional study by Li et al., children who had incident high weight or persistently high weight from birth to childhood had higher odds of childhood high BP than those who had persistently normal weight [54].

Generally, male sex has been considered a major risk factor in pediatric hypertension [55]. However, in the studies of overweight children and adolescents, the effect of sex has not been apparent [56, 57] In a large population-based study, BMI level and age were more significant than sex differences [58]. Other studies also reported that the effect of obesity on BP was greater with age [59, 60]. This may be related to hormonal changes during puberty.

Excessive salt intake has been associated with the development of obesity and high BP [61, 62], and the impact of excessive salt intake on BP is even more significant in obese individuals. A recent meta-analysis revealed that sodium intake was positively associated with BP in children and adolescents, especially more strongly in children with overweight and low potassium intake [63]. Another cross-sectional study in Portugal found that high sodium intake was associated with higher SBP in boys, and this was more pronounced in those who were overweight [64].

The lack of physical activity and sedentary behaviors, such as playing video games and watching television, are related to an increased risk of developing hypertension in overweight adolescents [43, 65, 66].

Recent studies have indicated a potential correlation between socioeconomic status (SES), hypertension, and BMI, particularly in the context of evolving social economies [67–70]. It is noteworthy that research in adults has revealed a paradox: in developing or developed nations, there is an inverse correlation between SES and hypertension, in contrast to under-developed countries. In Ghana, there was a positive association between SES and hypertension, which was partly due to differences in BMI [67]. Conversely, lower SES has been linked to higher hypertension rates in China and Iran [68, 69]. A study conducted in Hong Kong examined the relationship between SES and childhood obesity and hypertension; the findings revealed that children residing in the lowest SES neighborhoods were more likely to be underweight, overweight, or obese [70]. Additionally, the study indicated that having a less educated mother was associated with a higher risk of obesity and hypertension in children. This indicates the necessity for more sophisticated economic policy solutions to address these health issues.

### Treatment of hypertension in obesity

There are two main approaches to treating obesity-related hypertension. The first is to treat obesity and lower the BMI to below the 85th percentile, and the second is to treat BP itself. According to the published guidelines, lifestyle counseling for the DASH diet and moderate to vigorous exercise are recommended for all pediatric patients regardless of hypertension stage (Table 4) [2, 3].

**Table 4:** Lifestyle modifications for pediatric hypertension summarized from references

	2017 AAP guidelines [3]	2016 European Guidelines [2]
**General Recommendation**	Motivational interviewing may be a useful tool. Reducing stress	Implement of the behavioral change (Physical activity and diet) tailored to individual and family characteristics. Encourage parents/family participation. Encourage a smoke-free environment. Provide educational support and materials. Establish realistic goals. Develop a health-promoting reward system.
**Weight reduction**	Intensive weight-loss therapy for obese children with hypertension	Weight maintenance or gradual weight loss to achieve value < 85th percentile
**Physical activity**	Moderate to vigorous physical activity at least 3 to 5 days per week (30–60 min per session)	At least 60 min of activity per day, at least moderate-to-vigorous-intensity physical activity daily More activity provides additional health benefits. Aerobic mostly but with resistance components (3 times/week). Avoid more than 2 h of sedentary behavior per day. If uncontrolled stage 2 hypertension, avoid competitive sports.
**Diet**	**DASH diet** Fruits and vegetables 4–5 servings per day Low-fat milk products ≥ 2 servings per day Whole grains 6 servings per day Fish, poultry, and lean red meats ≤ 2 servings per day Legumes and nuts 1 serving per day Oils and fats 2–3 servings per day Added sugar and sweets (including sweetened beverages) ≤ 1 serving per day Dietary sodium < 2300 mg per d	Avoid intake of excess sugar, excess soft-sweetened drinks, saturated fat, and salt. Recommend fruits, vegetables, and grain products Limit sodium intake (< 2300 mg/daily).

*AAP* American Academy of Pediatrics, *DASH* Dietary Approaches to Stop Hypertension, *ESH*, European Society of Heart

Weight reduction is the key element in managing hypertension among overweight adolescents, considering the robust connection between adiposity and BP. For reducing BMI, diet and physical activity enhancement are essential [71]. In a meta-analysis study regarding nonpharmacological interventions and childhood obesity, combining diet and physical activity interventions was effective in reducing the risk of obesity in young children aged 0 to 5 years, while interventions focusing on physical activity alone were not effective in this age group [72]. However, interventions concentrating only on physical activity can reduce the risk of obesity in children aged 6 to 12 years and adolescents aged 13 to 18 years. There is no evidence that diet-only interventions are effective in these age groups, but interventions combining diet and physical activity may be effective.

In adults, weight loss has been associated with a reduction in BP. A meta-analysis of 18 studies revealed that losing 3–9% of body weight reduced SBP by 3 mmHg and DBP by 3 mmHg [73]. Another meta-analysis, encompassing 25 randomized controlled trials with 34 strata involving 4,874 individuals aged 37 to 66, explored weight reduction’s impact on BP. A reduction of 5.1 kg in body weight was associated with a decrease in SBP of 4.44 mmHg and DBP of 3.57 mmHg. Among those who lost more than 5 kg, the reduction in SBP was 6.63 mmHg; in DBP, it was 5.12 mmHg [74]. In obese adults, the greater the weight loss, the more significant the improvements in cardiovascular health and BP parameters [75, 76].

Several studies have investigated the BP-lowering effect of weight loss in obese or overweight children and adolescents [77–80]. The interventions included components such as diet, physical activity, education, and counseling and demonstrated a significant decrease in BP with weight loss. In a study that followed 5279 obese children for 32 months, both SBP and DBP decreased with lower BMI, and failure to treat obesity increased the risk of developing high BP levels [77]. In another study, a 12-week weight loss intervention in 115 obese children resulted in significant reductions in both SBP and DBP when weight was reduced [78].

#### Medical treatment for obesity

Drug therapy for the treatment of obesity in children is currently not approved. Furthermore, when considering drug therapy for children, a strong emphasis must be placed on assessing the risk-benefit ratio. Some drugs that have been considered for their potential effectiveness in treating obesity include amphetamines, fenfluramine, and dexfenfluramine.

Orlistat is an intestinal lipase inhibitor that reduces dietary fat and cholesterol absorption by 25%. The American Food and Drug Administration has approved it for obese adolescents over 12 years of age [81].

The obesity treatment drug liraglutide, approved for use in adults, has also been considered for use in pediatric diabetes. Recent results from a systematic review regarding its use for obesity treatment in pediatric populations indicate that it can be relatively safe and effective in children. In this study, there was a significant reduction in BMI and BMI standard deviation score, but the drop in SBP and DBP was not statistically significant [82]. However, this systematic review included studies of a small number of pediatric obese patients, an unclear method of BP measurement for the study subjects, and an unknown method of determining hypertension. More research is needed to further investigate the relationship between obesity treatment and hypertension.

A surgical approach may be considered in cases of severe obesity, but it is challenging to implement in children. In one prospective study, 242 adolescents aged 19 and under underwent weight-loss surgery, and the researchers discovered significant improvements in weight, cardiometabolic health, and weight-related quality of life three years after the procedure. The remission rate for elevated BP was 74% (95% CI, 64-84%) after three years [83].

#### Antihypertensive medication

**Fig. 1:**
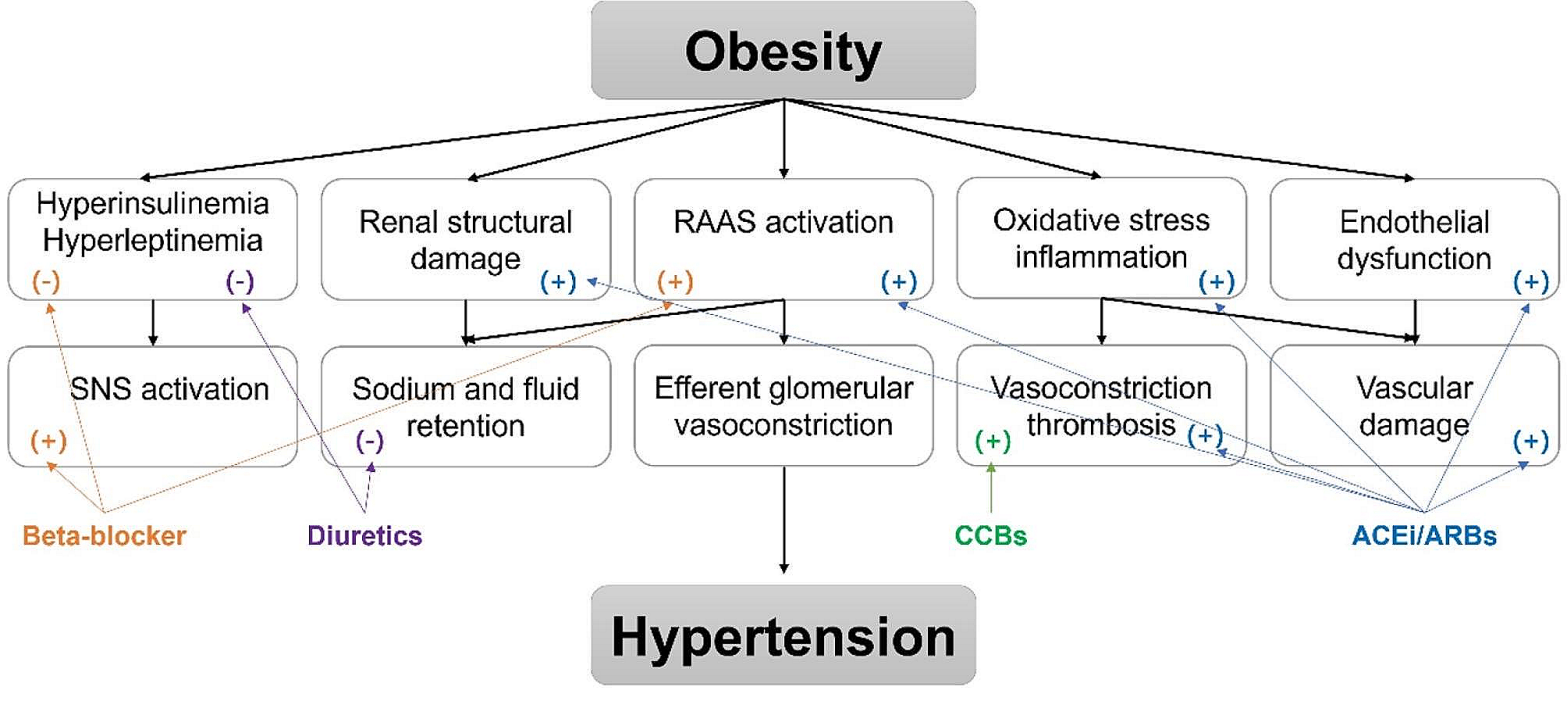
Mechanisms of hypertension in obesity and targets of antihypertensive drugs. *SNS* sympathetic nervous system, *RAAS* renin-angiotensin-aldosterone system, *ACEi* angiotensin-converting enzyme inhibitor, *ARB* angiotensin receptor blocker, *CCB* calcium channel blocker. (+) positive or protective effect, (-), negative effect. Figure adapted from *Hypertension in childhood obesity, E. Wühl, 2019*; adapted with permission; copyright John Wiley and Sons

According to the 2017 AAP guidelines, children with persistent or symptomatic hypertension despite attempts at lifestyle modification, stage 2 hypertension without clearly modifiable factors such as obesity, or any stage of hypertension associated with chronic kidney disease or diabetes should be started on a single drug at the low end of the dose range [3]. As is the case with non-obesity-related hypertension, BP should be managed to levels below the 90th percentile or 130/80 for children aged 13 years and older [3]. Drug choice should be targeted to the child’s underlying pathophysiology and the presence of concurrent disorders (Fig. 1).

Since one of the primary pathophysiologies of obesity-related hypertension is through activation of the RAAS system, ACE inhibitors or angiotensin receptor blockers (ARBs) may be appropriate as an initial agent for pharmacologic therapy for hypertension in children. There is some evidence to suggest the use of ACE inhibitors and ARBs as first-line agents in the obesity-linked primary hypertension population; in adults, these agents appear to reduce the incidence of new-onset diabetes and may increase insulin sensitivity [84]. Where these are not tolerated, calcium channel blockers are a reasonable alternative.

Given their known effects on glucose metabolism and insulin resistance, it is sensible to avoid using beta-blockers without vasodilatory capacity and thiazide diuretics [85]. In particular, diuretics can reduce intravascular volume and cardiac output and may stimulate the SNS and RAAS. They also have the potential to exacerbate insulin resistance and dyslipidemia and may increase glucose and uric acid levels, especially in obese individuals [86].

## Conclusion

In children, obesity is strongly associated with hypertension, and with the increase in obesity in recent years, hypertension has become an essential health condition in children. Several factors, including SNS and RAAS activity, cause the development of hypertension in obese children. Risk factors for hypertension in obesity include degree, duration, and distribution of obesity, patient age, hormonal changes during puberty, high-sodium diet, sedentary lifestyle, and SES. Treatment in obese children is a combination of treatment for obesity and hypertension. Treatment involves lifestyle changes, with weight loss being crucial to lowering BP.

## Data Availability

Not applicable.

## Abbreviations

AAP: American Academy of Pediatrics
ACE: Angiotensin-converting enzyme
ARB: Angiotensin receptor blocker
BMI: Body mass index
BP: Blood pressure
CI: Confidence interval
DASH: Dietary Approaches to Stop Hypertension
DBP: Diastolic blood pressure
KNHANES: Korean National Health and Nutrition Examination Survey
NHANES: National Health and Nutrition Examination Survey
NASH: National School Health Examination
RAAS: The renin-angiotensin-aldosterone system
SBP: Systolic blood pressure
SES: Socioeconomic status
SNS: Sympathetic nervous system
WC: Waist circumference
WHtR: Waist-to-height ratio

## References

[1] The fourth report on the. Diagnosis, evaluation, and treatment of high blood pressure in children and adolescents. Pediatrics. 2004;114(2 Suppl 4th Report):555 – 76. 15286277

[2] Lurbe E, Agabiti-Rosei E, Cruickshank JK, Dominiczak A, Erdine S, Hirth A, et al. 2016 European Society of Hypertension guidelines for the management of high blood pressure in children and adolescents. J Hypertens. 2016;34(10):1887–920. 27467768 10.1097/HJH.0000000000001039

[3] Flynn JT, Kaelber DC, Baker-Smith CM, Blowey D, Carroll AE, Daniels SR et al. Clinical practice Guideline for Screening and Management of High Blood pressure in children and adolescents. Pediatrics. 2017;140(3). 10.1542/peds.2017-1904 28827377

[4] Rabi DM, McBrien KA, Sapir-Pichhadze R, Nakhla M, Ahmed SB, Dumanski SM, et al. Hypertension Canada’s 2020 Comprehensive guidelines for the Prevention, diagnosis, Risk Assessment, and treatment of hypertension in adults and children. Can J Cardiol. 2020;36(5):596–624. 32389335 10.1016/j.cjca.2020.02.086

[5] Umemura S, Arima H, Arima S, Asayama K, Dohi Y, Hirooka Y, et al. The Japanese Society of Hypertension guidelines for the management of hypertension (JSH 2019). Hypertens Research: Official J Japanese Soc Hypertens. 2019;42(9):1235–481. 10.1038/s41440-019-0284-9 31375757

[6] Vos LE, Oren A, Uiterwaal C, Gorissen WH, Grobbee DE, Bots ML. Adolescent blood pressure and blood pressure tracking into young adulthood are related to subclinical atherosclerosis: the atherosclerosis risk in young adults (ARYA) study. Am J Hypertens. 2003;16(7):549–55. 12850388 10.1016/S0895-7061(03)00857-4

[7] Juhola J, Magnussen CG, Viikari JS, Kähönen M, Hutri-Kähönen N, Jula A, et al. Tracking of serum lipid levels, blood pressure, and body mass index from childhood to adulthood: the Cardiovascular Risk in Young finns Study. J Pediatr. 2011;159(4):584–90. 21514597 10.1016/j.jpeds.2011.03.021

[8] Allen NB, Siddique J, Wilkins JT, Shay C, Lewis CE, Goff DC, et al. Blood pressure trajectories in early adulthood and subclinical atherosclerosis in middle age. JAMA. 2014;311(5):490–7. 24496536 10.1001/jama.2013.285122 PMC4122296

[9] Theodore RF, Broadbent J, Nagin D, Ambler A, Hogan S, Ramrakha S, et al. Childhood to early-midlife systolic blood pressure trajectories: early-life predictors, Effect modifiers, and Adult Cardiovascular outcomes. Hypertension. 2015;66(6):1108–15. 26558818 10.1161/HYPERTENSIONAHA.115.05831 PMC4646716

[10] Cho H, Kim JH. Secular trends in hypertension and elevated blood pressure among Korean children and adolescents in the Korea National Health and Nutrition Examination Survey 2007–2015. J Clin Hypertens (Greenwich). 2020;22(4):590–7. 32175671 10.1111/jch.13842 PMC8029840

[11] Ye X, Yi Q, Shao J, Zhang Y, Zha M, Yang Q, et al. Trends in Prevalence of Hypertension and Hypertension Phenotypes among Chinese Children and adolescents over two decades (1991–2015). Front Cardiovasc Med. 2021;8:627741. 34046436 10.3389/fcvm.2021.627741 PMC8144307

[12] Jackson SL, Zhang Z, Wiltz JL, Loustalot F, Ritchey MD, Goodman AB, et al. Hypertension among youths - United States, 2001–2016. MMWR Morb Mortal Wkly Rep. 2018;67(27):758–62. 30001558 10.15585/mmwr.mm6727a2 PMC6047471

[13] Bell CS, Samuel JP, Samuels JA. Prevalence of hypertension in children. Hypertension. 2019;73(1):148–52. 30571555 10.1161/HYPERTENSIONAHA.118.11673 PMC6291260

[14] Song P, Zhang Y, Yu J, Zha M, Zhu Y, Rahimi K, et al. Global prevalence of hypertension in children: a systematic review and Meta-analysis. JAMA Pediatr. 2019;173(12):1154–63. 31589252 10.1001/jamapediatrics.2019.3310 PMC6784751

[15] Roulet C, Bovet P, Brauchli T, Simeoni U, Xi B, Santschi V, et al. Secular trends in blood pressure in children: a systematic review. J Clin Hypertens (Greenwich). 2017;19(5):488–97. 27982505 10.1111/jch.12955 PMC8031206

[16] Balasundaram P, Avulakunta ID. StatPearls [Internet]. StatPearls Publishing; Treasure Island (FL): Mar 8, 2023. Human Growth and Development. 33620844

[17] Hampl SE, Hassink SG, Skinner AC, Armstrong SC, Barlow SE, Bolling CF et al. Clinical practice Guideline for the evaluation and treatment of children and adolescents with obesity. Pediatrics. 2023;151(2). 10.1542/peds.2022-060640 36622115

[18] Levels, UNICEF/WHO/World Bank Group Joint Child Malnutrition Estimates. and trends in child malnutrition. New York (NY), Geneva and Washington (DC): United Nations Children’s Fund, World Health Organization and the World Bank Group; 2023.

[19] NCD Risk Factor Collaboration. Worldwide trends in body-mass index, underweight, overweight, and obesity from 1975 to 2016: a pooled analysis of 2416 population-based measurement studies in 128·9 million children, adolescents, and adults. Lancet. 2017;390(10113):2627–42. 29029897 10.1016/S0140-6736(17)32129-3 PMC5735219

[20] Fryar CD, Carroll MD, Afful J. Prevalence of overweight, obesity, and severe obesity among children and adolescents aged 2–19 years: United States, 1963–1965 through 2017–2018. NCHS Health E-Stats, Centers for Disease Control and Prevention. Updated January 29, 2021. Accessed April 22, 2021. www.cdc.gov/nchs/data/hestat/obesity-child-17-18/overweight-obesity-child-H.pdf External link (PDF, 352 KB).

[21] Kim JH, Moon JS. Secular trends in Pediatric overweight and obesity in Korea. J Obes Metab Syndr. 2020;29(1):12–7. 32188238 10.7570/jomes20002 PMC7118001

[22] Puri M, Flynn JT, Garcia M, Nussbaum H, Freeman K, DiMartino-Nardi JR. The frequency of elevated blood pressure in obese minority youth. J Clin Hypertens (Greenwich). 2008;10(2):119–24. 18256576 10.1111/j.1751-7176.2008.07285.x PMC8109979

[23] Chorin E, Hassidim A, Hartal M, Havakuk O, Flint N, Ziv-Baran T, et al. Trends in adolescents obesity and the Association between BMI and blood pressure: a cross-sectional study in 714,922 healthy teenagers. Am J Hypertens. 2015;28(9):1157–63. 25736450 10.1093/ajh/hpv007

[24] Wang X, Hu J, Huang S, Yang Z, Dong Y, Dong B, et al. Exploring overweight risk trajectories during Childhood and their associations with elevated blood pressure at late adolescence: a Retrospective Cohort Study. Hypertension. 2022;79(8):1605–13. 35094521 10.1161/HYPERTENSIONAHA.121.18714

[25] Sorof JM, Lai D, Turner J, Poffenbarger T, Portman RJ. Overweight, ethnicity, and the prevalence of hypertension in school-aged children. Pediatrics. 2004;113(3 Pt 1):475–82. 14993537 10.1542/peds.113.3.475

[26] Flechtner-Mors M, Neuhauser H, Reinehr T, Roost HP, Wiegand S, Siegfried W, et al. Blood pressure in 57,915 pediatric patients who are overweight or obese based on five reference systems. Am J Cardiol. 2015;115(11):1587–94. 25862158 10.1016/j.amjcard.2015.02.063

[27] Garrison RJ, Kannel WB, Stokes J 3rd, Castelli WP. Incidence and precursors of hypertension in young adults: the Framingham offspring study. Prev Med. 1987;16(2):235–51. 3588564 10.1016/0091-7435(87)90087-9

[28] Gentile CL, Orr JS, Davy BM, Davy KP. Modest weight gain is associated with sympathetic neural activation in nonobese humans. Am J Physiol Regul Integr Comp Physiol. 2007;292(5):R1834–8. 17218435 10.1152/ajpregu.00876.2006

[29] Lambert E, Sari CI, Dawood T, Nguyen J, McGrane M, Eikelis N, et al. Sympathetic nervous system activity is associated with obesity-induced subclinical organ damage in young adults. Hypertension. 2010;56(3):351–8. 20625075 10.1161/HYPERTENSIONAHA.110.155663

[30] Hall JE, da Silva AA, do, Carmo JM, Dubinion J, Hamza S, Munusamy S et al. Obesity-induced hypertension: role of sympathetic nervous system, leptin, and melanocortins. J Biol Chem. 2010;285(23):17271-6. 10.1074/jbc.R110.113175 PMC2878489 20348094

[31] Alvarez GE, Beske SD, Ballard TP, Davy KP. Sympathetic neural activation in visceral obesity. Circulation. 2002;106(20):2533–6. 12427647 10.1161/01.CIR.0000041244.79165.25

[32] Weyer C, Pratley RE, Snitker S, Spraul M, Ravussin E, Tataranni PA. Ethnic differences in insulinemia and sympathetic tone as links between obesity and blood pressure. Hypertension. 2000;36(4):531–7. 11040231 10.1161/01.HYP.36.4.531

[33] Dewan NA, Nieto FJ, Somers VK. Intermittent hypoxemia and OSA: implications for comorbidities. Chest. 2015;147(1):266–74. 25560865 10.1378/chest.14-0500 PMC4285080

[34] Ozata M, Ozdemir IC, Licinio J. Human leptin deficiency caused by a missense mutation: multiple endocrine defects, decreased sympathetic tone, and immune system dysfunction indicate new targets for leptin action, greater central than peripheral resistance to the effects of leptin, and spontaneous correction of leptin-mediated defects. J Clin Endocrinol Metab. 1999;84(10):3686–95. 10523015 10.1210/jcem.84.10.5999

[35] Mark AL, Correia ML, Rahmouni K, Haynes WG. Selective leptin resistance: a new concept in leptin physiology with cardiovascular implications. J Hypertens. 2002;20(7):1245–50. 12131511 10.1097/00004872-200207000-00001

[36] Engeli S, Sharma AM. The renin-angiotensin system and natriuretic peptides in obesity-associated hypertension. J Mol Med (Berl). 2001;79(1):21–9. 11327100 10.1007/s001090000144

[37] Bentley-Lewis R, Adler GK, Perlstein T, Seely EW, Hopkins PN, Williams GH, et al. Body mass index predicts aldosterone production in normotensive adults on a high-salt diet. J Clin Endocrinol Metab. 2007;92(11):4472–5. 17726083 10.1210/jc.2007-1088 PMC4428584

[38] Harte A, McTernan P, Chetty R, Coppack S, Katz J, Smith S, et al. Insulin-mediated upregulation of the renin angiotensin system in human subcutaneous adipocytes is reduced by rosiglitazone. Circulation. 2005;111(15):1954–61. 15837949 10.1161/01.CIR.0000161954.17870.5D

[39] Hall JE, do Carmo JM, da Silva AA, Wang Z, Hall ME. Obesity-induced hypertension: interaction of neurohumoral and renal mechanisms. Circ Res. 2015;116(6):991–1006. 25767285 10.1161/CIRCRESAHA.116.305697 PMC4363087

[40] Hall ME, do Carmo JM, da Silva AA, Juncos LA, Wang Z, Hall JE. Obesity, hypertension, and chronic kidney disease. Int J Nephrol Renovasc Dis. 2014;7:75–88. 24600241 10.2147/IJNRD.S39739 PMC3933708

[41] Hall JE, Brands MW, Dixon WN, Smith MJ. Jr. Obesity-induced hypertension. Renal function and systemic hemodynamics. Hypertension. 1993;22(3):292–9. 8349321 10.1161/01.HYP.22.3.292

[42] Wühl E. Hypertension in childhood obesity. Acta Paediatr. 2019;108(1):37–43. 30144170 10.1111/apa.14551

[43] Kelly RK, Magnussen CG, Sabin MA, Cheung M, Juonala M. Development of hypertension in overweight adolescents: a review. Adolesc Health Med Ther. 2015;6:171–87. 26543386 10.2147/AHMT.S55837 PMC4622556

[44] Nugent JT, Lu Y, Deng Y, Sharifi M, Greenberg JH. Effect measure modification by Birth Weight on the Association between overweight or obesity and hypertension in children and adolescents. JAMA Pediatr. 2023;177(7):735–7. 37155182 10.1001/jamapediatrics.2023.0799 PMC10167596

[45] Whelton PK, He J, Appel LJ, Cutler JA, Havas S, Kotchen TA, et al. Primary prevention of hypertension: clinical and public health advisory from the National High blood pressure education program. JAMA. 2002;288(15):1882–8. 12377087 10.1001/jama.288.15.1882

[46] Deng R, Lou K, Zhou S, Li X, Dong B, Ma J, et al. Associations of parental reproductive age and elevated blood pressure in offspring: an observational study. Front Pediatr. 2023;11:990725. 37063654 10.3389/fped.2023.990725 PMC10098010

[47] Babinska K, Kovacs L, Janko V, Dallos T, Feber J. Association between obesity and the severity of ambulatory hypertension in children and adolescents. J Am Soc Hypertens. 2012;6(5):356–63. 22995804 10.1016/j.jash.2012.08.002

[48] Chen L, Zhang J, Zhou N, Weng JY, Bao ZY, Wu LD. Association of different obesity patterns with hypertension in US male adults: a cross-sectional study. Sci Rep. 2023;13(1):10551. 37386040 10.1038/s41598-023-37302-x PMC10310720

[49] Janghorbani M, Aminorroaya A, Amini M. Comparison of different obesity indices for Predicting Incident Hypertension. High Blood Press Cardiovasc Prev. 2017;24(2):157–66. 28160265 10.1007/s40292-017-0186-3

[50] Ma C, Wang R, Liu Y, Lu Q, Lu N, Tian Y, et al. Performance of obesity indices for screening elevated blood pressure in pediatric population: systematic review and meta-analysis. Med (Baltim). 2016;95(39):e4811. 10.1097/MD.0000000000004811 PMC5265901 27684808

[51] Tao JM, Wei W, Ma XY, Huo YX, Hu MD, Li XF, et al. Diagnostic accuracy of anthropometric indices for discriminating elevated blood pressure in pediatric population: a systematic review and a meta-analysis. BMC Pediatr. 2022;22(1):19. 34983442 10.1186/s12887-021-03062-8 PMC8725266

[52] Tee JYH, Gan WY, Lim PY. Comparisons of body mass index, waist circumference, waist-to-height ratio and a body shape index (ABSI) in predicting high blood pressure among Malaysian adolescents: a cross-sectional study. BMJ Open. 2020;10(1):e032874. 31932391 10.1136/bmjopen-2019-032874 PMC7044891

[53] Kułaga Z, Świąder-Leśniak A, Kotowska A, Litwin M. Population-based references for waist and hip circumferences, waist-to-hip and waist-to-height ratios for children and adolescents, and evaluation of their predictive ability. Eur J Pediatr. 2023;182(7):3217–29. 37140701 10.1007/s00431-023-05001-4 PMC10353968

[54] Li C, Liu Z, Zhao M, Zhang C, Bovet P, Xi B. Weight status change from birth to childhood and the odds of high blood pressure among Chinese children. Front Public Health. 2023;11:1135994. 37089505 10.3389/fpubh.2023.1135994 PMC10116612

[55] Bucher BS, Ferrarini A, Weber N, Bullo M, Bianchetti MG, Simonetti GD. Primary hypertension in childhood. Curr Hypertens Rep. 2013;15(5):444–52. 23897423 10.1007/s11906-013-0378-8

[56] Nguyen S, McCulloch C, Brakeman P, Portale A, Hsu CY. Being overweight modifies the association between cardiovascular risk factors and microalbuminuria in adolescents. Pediatrics. 2008;121(1):37–45. 18166555 10.1542/peds.2007-3594 PMC3722048

[57] Hannon TS, Gupta S, Li Z, Eckert G, Carroll AE, Pratt JH, et al. The effect of body mass index on blood pressure varies by race among obese children. J Pediatr Endocrinol Metab. 2015;28(5–6):533–8. 25210760 10.1515/jpem-2014-0225 PMC9867844

[58] Koenigsberg J, Boyd GS, Gidding SS, Hassink SG, Falkner B. Association of age and sex with cardiovascular risk factors and insulin sensitivity in overweight children and adolescents. J Cardiometab Syndr. 2006;1(4):253–8. 17679813 10.1111/j.1559-4564.2006.05695.x

[59] Chiolero A, Cachat F, Burnier M, Paccaud F, Bovet P. Prevalence of hypertension in schoolchildren based on repeated measurements and association with overweight. J Hypertens. 2007;25(11):2209–17. 17921814 10.1097/HJH.0b013e3282ef48b2

[60] Koebnick C, Black MH, Wu J, Martinez MP, Smith N, Kuizon B, et al. High blood pressure in overweight and obese youth: implications for screening. J Clin Hypertens (Greenwich). 2013;15(11):793–805. 24119024 10.1111/jch.12199 PMC3849231

[61] Aparicio A, Rodriguez-Rodriguez E, Cuadrado-Soto E, Navia B, Lopez-Sobaler AM, Ortega RM. Estimation of salt intake assessed by urinary excretion of sodium over 24 h in Spanish subjects aged 7–11 years. Eur J Nutr. 2017;56(1):171–8. 26482149 10.1007/s00394-015-1067-y PMC5290043

[62] Wojcik M, Koziol-Kozakowska A, Obesity. Sodium Homeostasis, and arterial hypertension in children and adolescents. Nutrients. 2021;13(11). 10.3390/nu13114032 PMC8622119 34836287

[63] Leyvraz M, Chatelan A, da Costa BR, Taffe P, Paradis G, Bovet P, et al. Sodium intake and blood pressure in children and adolescents: a systematic review and meta-analysis of experimental and observational studies. Int J Epidemiol. 2018;47(6):1796–810. 29955869 10.1093/ije/dyy121

[64] Correia-Costa L, Cosme D, Nogueira-Silva L, Morato M, Sousa T, Moura C, et al. Gender and obesity modify the impact of salt intake on blood pressure in children. Pediatr Nephrol. 2016;31(2):279–88. 26420679 10.1007/s00467-015-3210-7

[65] Pardee PE, Norman GJ, Lustig RH, Preud’homme D, Schwimmer JB. Television viewing and hypertension in obese children. Am J Prev Med. 2007;33(6):439–43. 18022058 10.1016/j.amepre.2007.07.036

[66] Goldfield GS, Kenny GP, Hadjiyannakis S, Phillips P, Alberga AS, Saunders TJ, et al. Video game playing is independently associated with blood pressure and lipids in overweight and obese adolescents. PLoS ONE. 2011;6(11):e26643. 22069461 10.1371/journal.pone.0026643 PMC3206019

[67] Addo J, Smeeth L, Leon DA. Socioeconomic position and hypertension: a study of urban civil servants in Ghana. J Epidemiol Community Health. 2009;63(8):646–50. 19406743 10.1136/jech.2008.081828

[68] Luo T, Lin S, Zhang W, Li X, Wang Y, Zhou J, et al. Relationship between socioeconomic status and hypertension incidence among adults in southwest China: a population-based cohort study. BMC Public Health. 2024;24(1):1211. 38693482 10.1186/s12889-024-18686-5 PMC11064324

[69] Fateh M, Emamian MH, Asgari F, Alami A, Fotouhi A. Socioeconomic inequality in hypertension in Iran. J Hypertens. 2014;32(9):1782–8. 24979299 10.1097/HJH.0000000000000260

[70] Ip P, Ho FK, So HK, Chan DF, Ho M, Tso W, et al. Socioeconomic gradient in childhood obesity and hypertension: a Multilevel Population-based study in a Chinese Community. PLoS ONE. 2016;11(6):e0156945. 27258094 10.1371/journal.pone.0156945 PMC4892679

[71] Daniels SR, Hassink SG, Committee On N. The role of the Pediatrician in Primary Prevention of obesity. Pediatrics. 2015;136(1):e275–92. 26122812 10.1542/peds.2015-1558

[72] Brown T, Moore TH, Hooper L, Gao Y, Zayegh A, Ijaz S, et al. Interventions for preventing obesity in children. Cochrane Database Syst Rev. 2019;7(7):Cd001871. 31332776 10.1002/14651858.CD001871.pub4 PMC6646867

[73] Mulrow CD, Chiquette E, Angel L, Cornell J, Summerbell C, Anagnostelis B et al. Dieting to reduce body weight for controlling hypertension in adults. Cochrane Database Syst Rev. 2000(2):CD000484. 10.1002/14651858.CD000484 10796721

[74] Neter JE, Stam BE, Kok FJ, Grobbee DE, Geleijnse JM. Influence of weight reduction on blood pressure: a meta-analysis of randomized controlled trials. Hypertension. 2003;42(5):878–84. 12975389 10.1161/01.HYP.0000094221.86888.AE

[75] Rothberg AE, McEwen LN, Kraftson AT, Ajluni N, Fowler CE, Nay CK, et al. Impact of weight loss on waist circumference and the components of the metabolic syndrome. BMJ Open Diabetes Res Care. 2017;5(1):e000341. 28316795 10.1136/bmjdrc-2016-000341 PMC5337678

[76] Yang S, Zhou Z, Miao H, Zhang Y. Effect of weight loss on blood pressure changes in overweight patients: a systematic review and meta-analysis. J Clin Hypertens (Greenwich). 2023;25(5):404–15. 37141231 10.1111/jch.14661 PMC10184479

[77] Hagman E, Danielsson P, Elimam A, Marcus C. The effect of weight loss and weight gain on blood pressure in children and adolescents with obesity. Int J Obes (Lond). 2019;43(10):1988–94. 31152153 10.1038/s41366-019-0384-2

[78] Holm JC, Gamborg M, Neland M, Ward L, Gammeltoft S, Heitmann BL, et al. Longitudinal changes in blood pressure during weight loss and regain of weight in obese boys and girls. J Hypertens. 2012;30(2):368–74. 22157326 10.1097/HJH.0b013e32834e4a87

[79] Schaefer A, Winkel K, Finne E, Kolip P, Reinehr T. An effective lifestyle intervention in overweight children: one-year follow-up after the randomized controlled trial on Obeldicks light. Clin Nutr. 2011;30(5):629–33. 21514017 10.1016/j.clnu.2011.03.012

[80] Reinehr T, Schaefer A, Winkel K, Finne E, Toschke AM, Kolip P. An effective lifestyle intervention in overweight children: findings from a randomized controlled trial on Obeldicks light. Clin Nutr. 2010;29(3):331–6. 20106567 10.1016/j.clnu.2009.12.010

[81] Godoy-Matos AF, Guedes EP, Souza LL, Martins MF. Management of obesity in adolescents: state of art. Arq Bras Endocrinol Metabol. 2009;53(2):252–61. 19466218 10.1590/S0004-27302009000200017

[82] Gou H, Zhai Y, Guo J. Efficacy and safety of liraglutide for weight management in children and adolescents: a systematic review and meta-analysis of randomized controlled trials. Eur J Pediatr. 2023. 10.1007/s00431-023-05186-8 37672063

[83] Inge TH, Courcoulas AP, Xanthakos SA. Weight loss and health status after bariatric surgery in adolescents. N Engl J Med. 2016;374(20):1989–90. 27192678 10.1056/NEJMc1602007

[84] Elliott WJ, Meyer PM. Incident diabetes in clinical trials of antihypertensive drugs: a network meta-analysis. Lancet. 2007;369(9557):201–7. 17240286 10.1016/S0140-6736(07)60108-1

[85] Mancia G, Grassi G, Zanchetti A. New-onset diabetes and antihypertensive drugs. J Hypertens. 2006;24(1):3–10. 16331092 10.1097/01.hjh.0000194119.42722.21

[86] Brady TM. Obesity-related hypertension in children. Front Pediatr. 2017;5:197. 28993801 10.3389/fped.2017.00197 PMC5622310

